# Nematode-Applied Technology for Human Tumor Microenvironment Research and Development

**DOI:** 10.3390/cimb44020065

**Published:** 2022-02-21

**Authors:** Eric di Luccio, Satoru Kaifuchi, Nobuaki Kondo, Ryota Chijimatsu, Andrea Vecchion, Takaaki Hirotsu, Hideshi Ishii

**Affiliations:** 1 Hirotsu Bio Science Inc., Chiyoda-Ku, Tokyo 102-0094, Japan; e.diluccio@hbio.jp (E.d.L.); kaifuchi@hbio.jp (S.K.); kondo@hbio.jp (N.K.); 2 Department of Medical Data Science, Center of Medical Innovation and Translational Research, Osaka University Graduate School of Medicine, Suita, Yamadaoka 2-2, Osaka 565-0871, Japan; rchijimatsu@cfs.med.osaka-u.ac.jp; 3 Department of Clinical and Molecular Medicine, Santo Andrea Hospital, University of Rome “Sapienza”, Via di Grottarossa, 00189 Rome, Italy; andrea.vecchione@uniroma1.it

**Keywords:** nematode, microenvironment, cancer, research

## Abstract

Nematodes, such as *Caenorhabditis elegans*, have been instrumental to the study of cancer. Recently, their significance as powerful cancer biodiagnostic tools has emerged, but also for mechanism analysis and drug discovery. It is expected that nematode-applied technology will facilitate research and development on the human tumor microenvironment. In the history of cancer research, which has been spurred by numerous discoveries since the last century, nematodes have been important model organisms for the discovery of cancer microenvironment. First, microRNAs (miRNAs), which are noncoding small RNAs that exert various functions to control cell differentiation, were first discovered in *C. elegans* and have been actively incorporated into cancer research, especially in the study of cancer genome defects. Second, the excellent sense of smell of nematodes has been applied to the diagnosis of diseases, especially refractory tumors, such as human pancreatic cancer, by sensing complex volatile compounds derived from heterogeneous cancer microenvironment, which are difficult to analyze using ordinary analytical methods. Third, a nematode model system can help evaluate invadosomes, the phenomenon of cell invasion by direct observation, which has provided a new direction for cancer research by contributing to the elucidation of complex cell–cell communications. In this cutting-edge review, we highlight milestones in cancer research history and, from a unique viewpoint, focus on recent information on the contributions of nematodes in cancer research towards precision medicine in humans.

## 1. Introduction

Most nematode species live nonparasitic lives in soil and the ocean; however, many parasitic nematodes are also present [1]. Several nematodes, including human parasites, are closely related to human life, and while research on them has advanced, research on free-living animals has tended to be postponed. Enormous populations of nematodes are present in the soil, and account for 15% of earth biomass [2]. Cancer research began by considering the effects of its interaction with the environment before being deepened, and its complex mechanisms unraveled. Among them, nematodes have appeared in various points as research subjects or supporters that provide clues to cancer research. Particularly, as an extension of nematode research, these are: (1) the discovery of RNA interference, especially microRNAs, in cancer; (2) smell research objects and medical applications in cancer research; and (3) innovative applied methods for examining cell–cell interactions in the tumor microenvironment, all of which are discussed in this review article. We noted the milestones in cancer research and then focused on the advantages and discussed the usefulness of nematodes in the study of tumor microenvironment.

## 2. Milestones in Cancer Research

The cell theory was first described by Schleiden, Schwann, and Virchow [3]. Given that Rudolf Ludwig Karl Virchow (1821–1902), the founder of cellular pathology, who laid the foundations for cytopathology, comparative pathology (as a comparison of diseases common to humans with those common to animals), and anthropology, advocated his Latin motto “omnis cellula e cellula”, which means that every cell originates from a cell—the concept has been considered by many other current researchers that alterations in cell organization were the basis of disease [3,4]. He discovered the concept that only certain cells or groups of cells become sick, not the entire living body [3,4,5]. In the 1900s, the concept that tumors originate from other body parts was beginning to be debated. Considering the discussion that scrotal cancer, which was seen in factory chimney sweepers, is presumably due to repeated stimulation under the influence of the Industrial Revolution in Western countries. It was first described by Percival Pott in 1775 [6], and since then Virchow’s repetitive stimulus theory regarding cancer has emerged, and Fibiger’s work was a strong proof of Virchow’s theory; Fibiger received the Nobel Prize in Physiology and Medicine in 1926 because of his parasite carcinogenesis theory, for which he studied a type of parasite nematode called Spiroptera carcinoma (*Gongylonema neoplasticum*) [7]. Although, Fibiger’s Nobel Prize-winning parasite carcinogenesis theory is now believed to have been false, and a 2004 document investigating the 1926 Physiology and Medicine Award selection process has stated that it is easy to conclude that Fibiger’s Nobel Prize was wrong today; historically, it is invalid [7]. In this way, the royal road to the truth is to accumulate information, which has not been changed until modern life science. At that time, the main theories included “stimulation theory” and “predisposition theory,” which are discussed as the cause of cancer. Katsusaburo Yamagiwa succeeded in developing artificial cancer in 1915 by conducting experiments on the steady process of continuously rubbing coal tar on the ears of rabbits for over 3 years [7]. It remains supported that repetitive stimuli, especially the importance of inflammation, will contribute closely to the initiation, progression, and development of cancer, which suggests the importance of the cancer microenvironment. In 1931, Otto Warburg was awarded the Nobel Prize in Physiology and Medicine for his study on tumor metabolism and cell respiration, especially cancer cells [8]. The concept of metabolic reprogramming is now a hallmark of cancer [9,10]. DNA-sequencing techniques, which are now commonplace and incorporated into standard medical practice, began with the discovery of the Watson–Crick structure of DNA (double-helix structure) in the late 20th century [11], for which Watson and Crick received the 1962 Nobel Prize in Physiology and Medicine. Understanding the cancer microenvironment and communications between cells has become indispensable in understanding cancer overall. The study of the cancer genome has made great strides.

**Figure 1:**
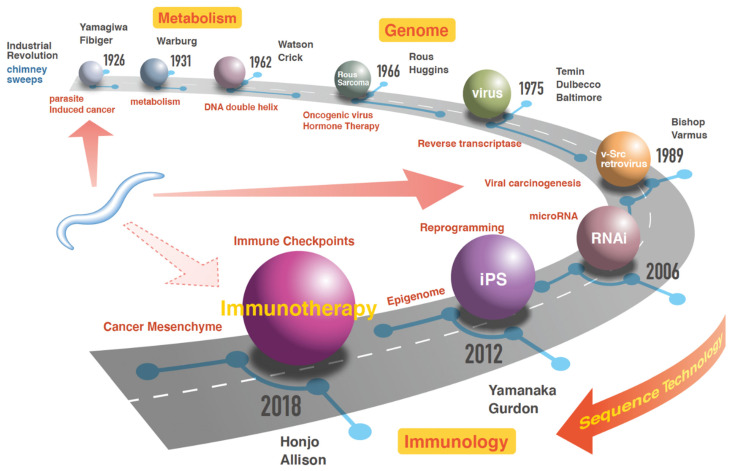
Historical overview of cancer research. Based on Virchow’s accomplishments on cellular pathology, the modern state of cancer research has been developed for over a century. With the background of the Industrial Revolution, cancer induction was studied by involving various stimuli, such as parasitic infections, nematode infections, or chemical substances, which were believed to induce inflammation in the epithelium. In 1962, DNA’s structure was elucidated, which opened the avenue to the current genome sequencing technology. Meanwhile, important discoveries were accumulated regarding viruses and their biochemistry. As a result of the discovery of *C. elegans* in the 21st century, miRNAs were discovered in human cancer. The iPS technology in regenerative medicine facilitated the study of reprogramming in cancer research. Immunotherapy is a current rewiring cancer treatment targeting the cancer microenvironment. The control of cell–cell communications in the cancer microenvironment is a critical issue in nematode technology. In a schema, sequential discoveries were illustrated according to the Nobel Prize in the field of cancer research. In the schema, the knowledge of nematode study induced innovation, which are depicted by three arrows. Detailed events are described in the text.

Rous has found non-epithelial malignancies that infect and develop not only when cancer cells are transplanted but also when substances extracted from cancer cells are injected [12]. This finding brought about the viral theory of cancer development. His work was ridiculed at the time; however, subsequent experiments proved his claim. He was awarded the 1966 Nobel Prize in Physiology and Medicine with Huggins, who discovered that hormones suppress the metastasis of certain cancers and showed for the first time that cancer can be controlled by chemicals [12]. Temin discovered reverse transcriptase in the 1970s and, along with Dulbecco and Baltimore, received the 1975 Nobel Prize in Physiology and Medicine [13]. Temin clarified how oncoviruses use reverse transcriptase to rewrite the genetic information of host cells. The discovery also urged a revision of the widely believed concept of Central Dogma, advocated by Watson and Crick, since other molecular biologists at the time believed that genetic information flows in only one direction, from DNA via RNA to protein. However, Temin demonstrated that in a type of tumor virus, reverse transcriptase is essential for transmitting genetic information toward DNA [13]. Varmus was awarded the Nobel Prize in Physiology and Medicine with Bishop for discovering the proto-oncogene tyrosine-protein kinase (c-Src), a human oncogene. Moreover, Varmus discovered that the cancer gene of a retrovirus has a cellular origin [14]. With the application of induced pluripotent stem (iPS) cells, Yamanaka was awarded the 2012 Nobel Prize in Physiology and Medicine, with co-winner Gurdon, for discovering that mature cells are reprogrammed and pluripotent. After this innovation of iPS technology [15], several studies have been conducted on the concept of reprogramming the properties of cells to regenerative medicine. Those concepts, i.e., metabolic reprogramming, which changes the metabolic mechanism from anaerobic to aerobic, and epigenetic reprogramming, which controls cell differentiation by regulating gene expression, have been applied in the diagnosis and treatment of human diseases. Recently, Honjo received the Nobel Prize in Physiology and Medicine in 2018 with Allison for the discovery of immune checkpoint inhibitors and their application to cancer treatment [16,17]. Honjo has been recognized for his seminal publication in 1992 describing a new molecule, which he termed programmed death-1 (PD-1), based on its functional role in mediating classical apoptosis in a T-cell hybridoma and hematopoietic progenitor cells [18]. Although tumor tissues contain carious components, including epithelial cancer cells, mesenchymal fibroblasts, blood vessels, and immune cells, given that cancer cells, but not noncancerous cells, harbor genetic alterations, much emphasis has been placed on the study of genetic alterations that can attenuate the function of tumor suppressor genes or induce the activation of tumor-promoting oncogenes [19]. Taken together, the study of genomic losses in cancer cells allowed the identification of microRNAs (miRNAs), which followed the discovery of miRNAs in nematodes. *C. elegans* has been crucial for miRNA research, that helped to unravel the role of miRNAs in cancer. Therefore, nematodes can be considered as a very useful tool to human cancer research (Figure 1).

## 3. MicroRNA

Fire and Mello were awarded the Nobel Prize in Physiology in 2006 for their discovery of RNA interference and gene silencing by double-stranded RNA [20]. Fire and Mellow, along with colleagues Xu, Montgomery, Kostas, and Sam Driver, translated small double-stranded RNAs (dsRNA) into proteins by disrupting mRNA with complementary sequences, which led to suppressing a specific gene. They found that dsRNA suppresses gene expression more efficiently than the previously reported RNA interference by single-stranded RNA. Since they needed short dsRNA, they suggested involving a catalytic process, and this hypothesis was substantiated by later studies [21,22]. The microRNAs lin-4 and lin-14 were discovered using genetic analysis of developmental timing mutants in *C. elegans* [23,24]. The study of the developmental timing pathway was pioneered by Brenner, Sulston and Horvitz on the genetics and cell lineage of *C. elegans* [25,26]. Horvitz defined several components of programmed cell death. *C. elegans* was key in understanding the general features of miRNA biology, which brings about the evaluation of the function of miRNAs [27]. In contrast, the microRNAs miR-15 and miR16 in cancer were discovered first in humans by positional cloning tumor suppressor genes of hematopoietic malignancies in cancer research of chromosome 13 [28], and the expression of these microRNAs was associated with the progression of chronic lymphocytic leukemia [29]. A study on lung cancer has shown that the expression of the microRNA let-7 in human solid tumors, such as lung cancers, was decreased and associated with shortened postoperative survival [30]. The study has indicated that the expression of miRNAs successfully classifies poorly differentiated tumors using miRNA expression profiles, whereas messenger RNA profiles are highly inaccurate, suggesting the potential of miRNA profiling in cancer diagnosis [31].

## 4. Smell Research

It is common knowledge that some animals have sensory abilities superior to those of humans, such as smelling, and animal use will fill the gap in achieving detection of phenomena via smelling [32]. In a study by Lo et al., memory tests of *Canis familiaris*, *Rattus norvegicus*, and *Homo sapiens* indicated that dogs were superior to rats and that dogs and rats were superior to humans [33]. The study has suggested that the relatively poor performance of humans contrasts with high recognition memory for odors, suggesting that humans complement their low sensory abilities with intelligence and emotions [33]. Therefore, using animals will be better for objective testing. Incidentally, there have been some subjective opinions that patients with cancer have a peculiar odor; however, there was no way to objectively investigate this theory. Attempts were made to use animals to test this theory as objectively as possible. To examine the ability of beagle dogs to discriminate fresh biopsy and discharge samples from patients with cervical cancer, which is based on the impression of clinical doctors that cervical cancer with discharges might express any odors, a double-blinded procedure was performed. The results indicated that trained dogs seemed useful as a noninvasive alternative method for identifying patients with cervical cancer [34]. Moreover, another study has indicated that canine olfaction can detect liquid samples from breast cancer and colorectal cancer cell cultures, although dogs could not discriminate the odor of metabolic wastes between breast and colorectal cancers [35], suggesting that such animals sense some odors that have not been characterized so far. Although dogs may be considered good candidates for scent test detectors, the cost for training and maintenance is relatively high, which inhibits repeats and requires several examinations a day to confirm its reproducibility, which humps a large-scale study [32].

Nematodes may be an ideal tool to assess odorants from samples, such as urines of patients with cancer, and study uncharacterized mechanisms that will reflect tumor microenvironments. Studies have indicated that *C. elegans* could discriminate urine samples from patients with cancer from those obtained from healthy individuals [36]. The receiver operating characteristic (ROC) analysis has indicated that tests using *C. elegans* had a higher diagnostic ability than those using classical tumor markers; moreover, *C. elegans* showed a significant difference in behavior before and after tumor removal, suggesting that the *C. elegans* test will be useful in monitoring patients postoperatively [37]. Moreover, a relatively large study involving 180 urine samples from patients with gastrointestinal cancer and 76 samples from healthy participants has demonstrated that gastrointestinal cancer screening test has a high sensitivity, with a significant value of 0.80 in the ROC analysis, even in early-stage cancers [38]. Furthermore, a nationwide study group comprising high-volume centers throughout Japan to collect patients with pancreatic cancer reported that an open-label study involving 83 cases (stage 0–IV) of pancreatic cancer showed the efficacy of the *C. elegans* test to detect pancreatic cancer; a blinded study on 28 cases conducted by comparing patients with very early stage pancreatic cancer indicated that preoperative urine samples had a significantly higher chemotaxis index than postoperative samples in patients with pancreatic cancer; using the changes in the preoperative and postoperative chemotaxis index, this method had a higher sensitivity for detecting early pancreatic cancer than existing diagnostic markers, suggesting the rationales for the clinical application of *C. elegans* in the early diagnosis of pancreatic cancer [39]. In contrast, a study on the mechanism of genetically engineered mice indicated that the *C. elegans* test detected the urine of oncogenic KrasG12D mouse model, which is frequently mutated and activated in pancreatic cancer in humans, whereas the role of mouse c-Met, a receptor of hepatocyte growth factor, was not detected [40], suggesting that the downstream products of mouse KrasG12D is involved in the chemotaxis or olfactory behavior response alteration in *C. elegans*.

Many parasitic nematodes actively search for hosts to infect by using volatile chemical cues [41]. By understanding the olfactory signals of free-living nematodes as conventional research tools, we will be able to apply the knowledge to prevent infection by parasitic nematodes in humans. Eventually, the study of circuit mechanisms has allowed the identification of substances, including odorants, gases, and pheromones, that *C. elegans* respond to [41]. It shows that chemosensory neurons of *C. elegans* include: amphid wing C (AWC), which functions as an attraction by sensing odors, temperatures, carbon dioxide, salt, osmotic pressure, and pH; AWA olfactory neuron, which functions as an attraction by sensing odors; ASH sensory neuron, which mediates avoidance by sensing odors, soluble chemicals, and mechanical and osmotic stimuli; BAG neuron, which functions as avoidance (adults) or attraction (daughters) by sensing carbon dioxide and oxygen; and ADL neuron, which functions as avoidance by sensing odors and pheromones [41] (https://www.wormatlas.org) (accessed on 1 November 2021). The proposed models of microcircuit motifs present in the olfactory system of *C. elegans* indicate two stages. First, the feedback-inhibition regulatory system can elicit odor adaptation [42]. In the absence of an odor, AWC olfactory neurons will release neuropeptide-like protein 1 (NLP-1), which binds the neuropeptide receptor resemblance-11 (NPR-11) on the surface of AIA interneurons to inhibit their activity. In contrast, in the presence of an odor, AWC activity is suppressed, resulting in a decrease in NLP-1 signaling and leads AIA to release insulin-related 1 (ins-1), which inhibits AWC [42]. Second, the reciprocal inhibition system can modulate feedings in an odor environment [43]. In the presence of attractive odors, nematodes increase their feeding. As a mechanism, attractive odorants, such as diacetyl, are sensed by AWA neurons and cause the release of serotonin (5-hydroxytryptamine, 5-HT) from NSM neurons. 5-HT binds MOD-1 (Modulation Of locomotion Defective), a serotonin-gated chloride channel on RIM and RIC interneurons, resulting in inhibition and an increase in feeding. In contrast, the presence of repulsive odors decreases feeding caused by repellents, such as quinine, or high concentrations of isoamyl alcohol, which are sensed by ASH neurons and promote the release of octopamine and tyramine from RIM and RIC. Octopamine and tyramine bind to the tyramine receptor (SER-2) on NSM neurons and inhibit serotonin release [43]. As such, uncharacterized substances, including some volatiles, may be involved in the response to stimuli in nematodes [36,39,44]. A study on urine samples from patients with pancreatic cancer showed unique patterns of volatile organic compounds, suggesting that they are useful in distinguishing between cancer and inflammation in the pancreas [45]. Moreover, a study on pancreatic ductal adenocarcinoma has indicated that acetone, 2-pantanone, 4-methyl-2-heptanone, D-limonene, and levomenthol were possible volatile organic compounds and metabolite biomarkers in urine, though both chronic pancreatitis and pancreatic ductal adenocarcinoma were investigated [46]. Furthermore, another study has suggested several candidate volatile organic compounds, including 2-octonone and pentanal, as these compounds increased in the urine of patients with prostate cancer compared with those in healthy controls [47].

Recent studies of lung cancer have indicated that volatile organic compounds in breath are potentially associated with disease progression, suggesting its usefulness as a biomarker [48,49,50,51,52,53]. A study on the linkage between volatile organic compounds and gene mutations of KRASV12 and TP53 has indicated that genetic changes lead to detectable differences in levels of specific volatile organic compounds in cell culture experiments, suggesting that breath analysis can be used for detecting cancers [54]. Volatile organic compounds may be involved in other mutations observed in cancer [55].

**Figure 2:**
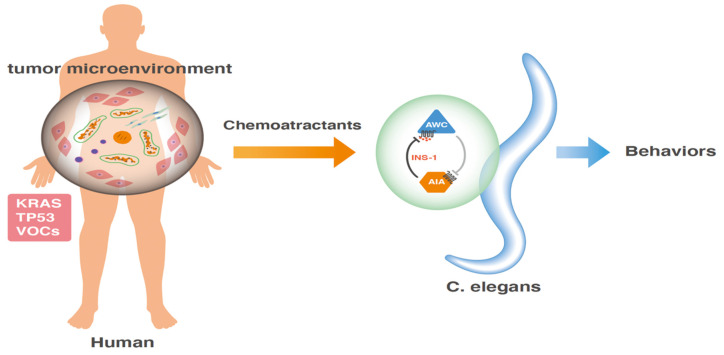
Schema for the nematode scent test of cancer. Given that cancer is a genetic disease harboring the accumulation of several mutations of malignant phenotype-promoting oncogenes and tumor suppressor genes. In pancreatic cancer, mutations in KRAS and TP53 occur frequently, which stimulate the downstream signals in cancer cells, influencing the surrounding mesenchymal fibroblasts, vessels, neural cells, and immune cells in the tumor microenvironment. Studies have indicated that *C. elegans* respond differentially to the presence or absence of tumors by sensing liquid samples, such as urine from patients with cancer. As a mechanism, volatile organic compounds, the production of which was elicited in the influence of genetic mutations of KRAS and TP53, may be involved in the behavior reaction by stimulating the neural system in nematodes. Detailed events are described in the text.

The gene mutation-related mechanism by which nematodes sense the smell of cancer is interesting. Substances that can stimulate nematode nerve cells may be released from cancerous tissues under the control of KrasG12D, but not Met activation, which elicited a response from nematode nerve cells according to animal experiments [40]. Metabolites located downstream of KRASG12D may be involved. However, a clinical sequence study has indicated that KRAS is mutated in more than 90% of cases of pancreatic cancer, with frequent associations with other mutations, such as mothers against decapentaplegic homolog 1 (SMAD1) family in the transforming growth factor beta 1 (TGF-β) pathway, and tumor suppressor genes, including tumor protein P53 (TP53) and cyclin-dependent kinase inhibitor 2A (p16Ink4a) [56]. Recently, a study on the metabolism in pancreatic cancer has demonstrated that pancreatic cancer cells rely on the distinct pathway in which glutamine supports pancreatic cancer growth through a KRAS-regulated metabolic pathway [57]. Glutamine is converted to oxaloacetate by aspartate transaminase (GOT1), and oxaloacetate is converted further into malate and then pyruvate, and this metabolic pathway is associated with an increase in the NADPH/NADP+ ratio, resulting in the maintenance of the cellular redox state [57]. Taken together, it appears that various metabolic pathway abnormalities occur downstream of the KRAS mutation in pancreatic cancer, which results in the generation of substances that affect the odorant behaviors of nematodes. Further studies undoubtedly will be necessary to further understand the mechanism of *C. elegans* sensing to develop an efficient innovative tool (Figure 2).

## 5. Innovative Method for Studying the Tumor Microenvironment

Recently, animal models, including nematodes, are used for basic studies for medical applications, such as mechanism studies and drug discovery, as summarized in [32]. Here, we focused on another application to study the tumor microenvironment using *C. elegans*. Recent studies have reported, in general, the importance of invadosomes, including podosomes and invadopodia, which are involved in cell–cell interactions via specialized F-actin-based adhesive structures formed as cell protrusions at sites of cell–extracellular matrix contacts on the ventral membrane of various cell types in tumor tissues [58]. Invadosomes are referred to as podosomes when they are found in normal cells and invadopodia when they are found in cancer cells. In this review, we discussed both, considering that common mechanisms are shared between them [59,60]. In vivo invadosome homologs have been reported in developmental model systems, including *C. elegans* [61]. The phenomenon of invasion occurs during both physiological and pathological processes. The formation of invadosomes is observed in various cells, including vascular cells, monocytic cells, osteoclasts, cancer cells, fibroblasts, and cancer-associated fibroblasts, which are transformed by oncogenic signals on almost all life processes in different stages of embryonic and tissue development, wound-healing, inflammation, and cancer invasion and metastasis, which are characteristics of the tumor microenvironment [60,62]. The structures of invadosomes were first discovered in a study of chicken embryo fibroblasts transformed using v-Src, a viral oncogene found in Rous sarcoma virus (RSV) [63]. The small size and transparent nature of *C. elegans* offer an important feature of being able to visualize invasive protrusions in vivo, which can address the issues in observing in higher organisms. Thus, *C. elegans* is often used as a model system in studies of developmental processes [61]. The genome of *C. elegans* encodes orthologs of most components implicated in invadosome formation or function, including Src [64,65]. Studies have reported that one exception to the structural components observed in *C. elegans* is cortactin, a key regulator of invadosome formation in cancer cells in vitro, suggesting that the common mechanism is shared over species [64,66,67], showing the valuable significance of nematode application to human life science. The first study that has used *C. elegans* has indicated that in vivo screening of genes regulating invadopodia allowed the identification of genes promoting invadopodia function in vivo—cell division control protein 42 homolog (CDC42) and Rab GDP dissociation inhibitor 1 (Gdi1)—which are involved in the direct control of invadopodia formation. The aforementioned results clarified the notion that invadopodia formation requires the integration of distinct cellular processes coordinated by an extracellular cue [68]. For the screening of cell–cell interactions and cytokines and chemokines, especially volatile organic compounds secreted and contained in the cancer microenvironment and expectedly sensed by nematodes as the cancer screening, the nematode system is expected to produce new results that have never been seen.
